# The formation and accumulation of protein-networks by physical interactions in the rapid occlusion of laticifer cells in rubber tree undergoing successive mechanical wounding

**DOI:** 10.1186/s12870-018-1617-6

**Published:** 2019-01-07

**Authors:** Minjing Shi, Yan Li, Shunnan Deng, Dongdong Wang, Yueyi Chen, Shuguang Yang, Jilin Wu, Wei-Min Tian

**Affiliations:** 1 Institute of Rubber Research, Chinese Academy of Tropical Agricultural Sciences, Danzhou, 571737 Hainan People’s Republic of China; 2 Key Laboratory of Biology and Genetic Resources of Rubber Tree, Ministry of Agriculture, Danzhou, 571737 Hainan People’s Republic of China; 3 Dehong Vocational College, Mangshi, Dehong State, 678400 Yunnan People’s Republic of China

**Keywords:** *Hevea brasilensis* Muell. Arg., Laticifer cell, Protein-network, Protein interaction, Cytoskeleton, Mechanical wounding

## Abstract

**Background:**

Although the wound response of plants has been extensively studied, little is known of the rapid occlusion of wounded cell itself. The laticifer in rubber tree is a specific type of tissue for natural rubber biosynthesis and storage. In natural rubber production, tapping is used to harvest the latex which flows out from the severed laticifer in the bark. Therefore, study of the rapid wound-occlusion of severed laticifer cells is important for understanding the rubber tree being protected from the continuously mechanical wounding.

**Results:**

Using cytological and biochemical techniques, we revealed a biochemical mechanism for the rapid occlusion of severed laticifer cells. A protein-network appeared rapidly after tapping and accumulated gradually along with the latex loss at the severed site of laticifer cells. Triple immunofluorescence histochemical localization showed that the primary components of the protein-network were chitinase, β-1,3-glucanase and hevein together with pro-hevein (ProH) and its carboxyl-terminal part. Molecular sieve chromatography showed that the physical interactions among these proteins occurred under the condition of neutral pH. The interaction of β-1,3-glucanase respectively with hevein, chitinase and ProH was testified by surface plasmon resonance (SPR). The interaction between actin and β-1,3-glucanase out of the protein inclusions of lutoids was revealed by pull-down. This interaction was pharmacologically verified by cytochalasin B–caused significant prolongation of the duration of latex flow in the field.

**Conclusions:**

The formation of protein-network by interactions of the proteins with anti-pathogen activity released from lutoids and accumulation of protein-network by binding to the cytoskeleton are crucial for the rapid occlusion of laticifer cells in rubber tree. The protein-network at the wounded site of laticifer cells provides not only a physical barrier but also a biochemical barrier to protect the wounded laticifer cells from pathogen invasion.

## Background

Plants survive in their sessile condition by coping with numerous environmental stresses, which includes the inevitable mechanical wounding caused by abiotic and biotic factors such as wind, herbivorous insects and animals. Therefore, plants have evolved sophisticated mechanisms to promptly respond to wounding, rapidly heal the tissue and induce defence strategies to prevent microbial infections [1–3]. Hormonal signals, especially jasmonate signalling [4–7] and other chemical signals such as nitric oxide [8, 9], hydrogen peroxide [10, 11], cell wall-derived oligogalacturonides (OGs) [12], peptide systemin [13, 14] and physical signals as hydraulic pressure and electrical signals [15, 16] link the wound perception to responsive strategies such as accumulation of metabolites with anti-pathogen or anti-digestive activities locally and systemly [17–22], activation of programmed cell death near the wounded sites [23] and the formation of wound periderm for a long time [24]. In such processes as potato wound-healing, it is the cell regeneration other than the healing of wounded cell itself [25].

The rapid healing of wounded cell itself occurs in such case as the occlusion of severed sieve tube by callose accumulation at sieve plates and possibly, protein plugging of the sieve pores when the phloem is occasionally injuried [26]. Laticifer is another tubing structure like as the sieve tube, and is constituted of living cells containing the latex, but our understanding about its role in plant still has remained limited [27–30]. In the latex-bearing plants, the rubber tree (*Hevea brasiliensis* Muell. Arg.) is the most important cultivated plant that produces commercial natural rubber in latex. For exploiting the latex, laticifers in the trunk bark of rubber tree are severed by tapping (mechanical wounding). Usually, the occlusion of severed laticifers in the rubber trees occurs after tapping in natural rubber production. Only several hours are taken to plug the wounded laticifer cells resulting in the cessation of tapping-caused latex flow. Therefore, successive tappings are required to cut off the plug materials and repeatedly return the latex flow from the severed laticifer under high turgor pressure of laticifers [31]. In this way, the rubber tree suffers ten tappings each month with a 2-day interval. However, 2 days are not sufficient for the formation of wound periderm that is an important defence tissue [32, 33].

It thus has long believed that rubber coagula formation caused by the fractured lutoids (a special lysosomal microvacuole in latex) contributes to the rapid occlusion of wounded laticifers based on the electron microscopic observations [34]. There are two stages of rubber coagula formation, the rubber particle (a special monolayer membrane organelle in latex) aggregation triggered by factors from lutoids and spontaneously membrane fusion of the aggregated rubber particles. Various kinds of factors which are mainly localized in lutoids, such as cationic proteins and bivalent cation [35], acid hydrolases and oxidoreductases [36], lectins [37, 38] and polyphenoloxidase (PPO) [39–41] are suggested to mediate the rubber particle aggregation that results in the rubber coagula formation. Alternatively, the cessation of latex flow has nothing to do with the rubber coagula formation because of large number of rubber particle aggregates with intact membrane and electron dense protein network instead of rubber coagula at the end of severed laticifer soon after the latex flow stops [42]. The present study reveals the formation of protein network by interactions of the proteins with anti-pathogen activity released from lutoids and accumulation of protein-network by binding to the cytoskeleton by using cytological and biochemical techniques, which is essential for the severed laticifer occlusion against the relatively high turgor pressure of laticifers.

## Materials and methods

### Plant materials and treatments

Four-year-old regularly tapped rubber trees of clone CATAS7–33-97 were grown in the Experimental Farm of the Chinese Academy of Tropical Agricultural Sciences (CATAS) in Danzhou city, Hainan province, P. R. China. Ten of the regularly tapped trees were selected to observe the plug formation and accumulation at the severed laticifers. Fresh bark samples at tapping cuts were separately collected from the regularly tapped trees at 0 and 5 min after tapping, immediately after the termination of latex flow and at 24 h after the termination of latex flow.

To obtain the purified lutoids, within 5–20 min after tapping, fresh latex samples were collected from 10 regularly tapped trees and mixed in ice-chilled tubes. The latex was fractionated into the floating rubber layer, C-serum (latex cytosol) and the bottom deposits (crude lutoids) by centrifugation at 38,760×g for 1 h at 4 °C. Only the bottom fraction was collected and re-suspended in ice-cold washing solution (50 mM Tris-HCl, 400 mM mannitol, pH 7.4) at a ratio of 1:10 (*w*/*v*), incubated for 10 min on ice, and then ultracentrifuged at 17,230×g for 30 min at 4 °C. After washing three times with washing buffer, the clear bottom fraction (purified lutoids) was used to prepare B-serum and lutoid debris by treatment with freeze-thaw cycles.

To test the effect of cytochalasin B on the duration of latex flow in the field, twenty regularly tapped trees of rubber tree clone CATAS7–33-97 were selected based on the duration of latex flow and divided into 2 groups. The average duration of latex flow was approximately the same between the groups. Ten trees in one group were treated for 48 h with 0.1% cytochalasin B in dimethyl sulfoxide (DMSO) solution (*w*/*v*), and as a control, 10 trees from the other group were treated with DMSO solution only. Thereafter, five successive tappings were performed for all the trees with a tapping system of S/2 d/3 (tapped the bark with a half of girth and once every 3 days), and the duration of latex flow was measured for each tapping. The experiments were conducted as three biological replicates in August, September and October.

### Light microscopy

The in situ detection of proteinaceous materials followed Tian and Hao [43]. The fresh bark samples were fixed in 4% glutaraldehyde in 0.1 M phosphate buffer solution at pH 7.2 for 24 h at room temperature and then washed 3 times with phosphate buffer solution, dehydrated in an increasing ethanol series, and embedded in paraffin. Sections (15 μm thickness) were cut with a microtome and stained with mercury-bromophenol blue. The proteinaceous material was clear blue under a microscope (DMLB, Leica, Germany).

### Electron microscopy

Fresh bark samples were immediately immersed in chilled 6% glutaraldehyde in 0.1 M phosphate buffer (pH 7.2) for 2 h and then cut into the correct sizes, fixed in the glutaraldehyde solution at 4 °C for 22 h and post-fixed in 2% OsO4 in 0.1 M phosphate buffer (pH 7.2) for 6 h at room temperature. The bark samples were dehydrated in ethanol as above and embedded in Epon 812 resin. Ultrathin sections were then cut with a LKB-V microtome, stained in uranyl acetate and lead citrate, and examined in a JEM100CX-II electron microscope, as described by Hao et al. [42].

### Triple immunofluorescence histochemical localization

The antibody preparation described as Wang et al. [44]. The 15–20 AA length-specific peptides of hevein, chitinase and β-1,3-glucanase were synthesized and coupled with bovine serum albumin (BSA) for that of hevein and with keyhole limpet hemocyanin (KLH) for that of chitinase and β-1,3-glucanase, and subsequently were respectively used to immunize New Zealand rabbits, chicken and mice. The fresh bark samples were fixed in a chilled fixative solution (4% formaldehyde and 3% acetic acid in 0.9% sodium chloride) for 8 h at 4 °C and then washed with 0.9% sodium chloride solution, dehydrated in an increasing ethanol series, and embedded in paraffin. Sections (15 μm thickness) were cut with a microtome. The sections were soaked in 100 mM ammonium chloride in TBS (20 mM tris-HCl, 500 mM sodium chloride, pH 7.2) for 60 min and washed three times with TBS containing 0.02% Tween-20 for 30 min. Thereafter, the sections were soaked in TBS containing 10 mM glycine for 30 min and blocked in TBS containing 10% nonfat dry milk overnight at 4 °C. The sections were incubated with a mixture of mouse anti-glucanase, chicken anti-chitinase, and rabbit anti-hevein (1:5 ratio of primary antibody diluted to TBS containing 10 mM glycine and 10% nonfat dry milk) in a moist chamber at 37 °C for 2 h. Then, the sections were rinsed three times with TBS containing 10% nonfat dry milk and 10 mM glycine for 60 min and incubated with a mixture of rhodamine Red-X(RRX)-conjugated goat anti-mouse IgG (PIERCE, America), aminomethylcoumarin acetate (AMCA)-conjugated goat anti-chicken IgG (PIERCE, America), and fluorescein isothiocyanate (FITC)-conjugated goat anti-rabbit IgG (SIGMA, America) (1:50 dilution) in a moist chamber at 37 °C for 2 h. After rinsing three times with TBS for 30 min, the sections were examined under a laser scanning confocal microscope (LSCM 510; ZEISS, Germany) at emission wavelength 488 nm for FITC, 543 nm for RRX, and 405 nm for AMCA. Control slides were prepared in the same way, except that pre-immune serum was used instead of the primary antibody.

### Tricine-SDS-PAGE and western blotting

Tricine-sodium dodecyl sulphate-polyacrylamide gel electrophoresis (Tricine-SDS-PAGE) was performed as described by Schagger et al. [45] and Shi et al. [46]. Polyacrylamide concentrations in the stacking gel and separating gel were 4 and 14%, respectively. Electrophoresis was conducted at a voltage of 30 V in the stacking gel and a constant voltage of 100 V for the separating gel. The gels were immersed in a fixer containing 0.5% glutaraldehyde and 30% alcohol for 30 min and stained with 0.1% Coomassie Brilliant Blue R-250.

For western blotting analysis, proteins were electrophoretically transferred from Tricine-SDS-PAGE gel to a polyvinylidene fluoride (PVDF) membrane. The electrode solution was composed of 20 mM Tris base, 150 mM glycine and 20% (*v*/v) methanol [47]. The transferred PVDF membrane was fixed with 0.2% (v/v) glutaraldehyde in TBS buffer(20 mM Tris+ 0.5 M NaCl, pH 7.5) for 45 min and washed three times with TBS buffer for 30 min. The fixed PVDF membranes were blocked and then incubated for 2–3 h with a polyclonal mouse anti-glucanase, chicken anti-chitinase, and rabbit anti-hevein at room temperature. The localization of alkaline phosphatase (AP) conjugated antibodies (PIERCE, American) was performed using the BCIP/NBT kit from TIANGEN Biotech Co., Ltd. (China), according to the manufacturer’s instructions. The controls were performed using a pre-immune serum instead of immune serum.

### Extraction of proteins from lutoids with different buffers

The clear bottom fraction (purified lutoids) was respectively re-suspended into four types of buffer: 50 mM Tris-HCl buffer with pH of 7.3 and 7.5, and 50 mM sodium acetate buffer with pH of 5.0 and 5.5. The re-suspensions were subjected to freeze-thaw cycles (− 20 and 37 °C) to release their fluid inclusions (B-serum) and then centrifuged at 38,760×g for 60 min at 4 °C. Proteins in the supernatant (upper fraction) were determined by the Bradford assay [48] using BSA as the standard. 8 μg of the soluble protein was loaded per lane in the gel of Tricine-SDA-PAGE. The precipitates (bottom fraction of lutoid membrane debris) were collected and washed at least 4 times with the corresponding buffer by centrifugation until no proteins were detected in the washing solution by Tricine-SDS-PAGE. To analyse the protein compositions in lutoid membrane debris that were washed with different buffers, 0.01 g of lutoid membrane debris was suspended in 80 μL of ultrapure water and then mixed with 80 μL of loading buffer (2×), boiled for 20 min at 95 °C and centrifuged at 13,680×g for 10 min at 25 °C. The supernatant was collected. 10 μL of the supernatant was loaded per lane in the gel of Tricine-SDS-PAGE.

### Purification of primary proteins in B-serum (inclusions of lutoids)

The clear lutoids without addition of any buffers were subjected to three freeze-thaw cycles (− 20 and 37 °C) to release the protein inclusions in lutoids and then centrifuged at 38,760×g for 60 min at 4 °C. The supernatant (B-serum) was collected.

Proteins in the B-serum were fractionated with 0–65%, 65–85% and 85–95% (NH_4_)_2_SO_4_ saturation in sequence [49]. The protein pellets were recovered by centrifugation at 38,760×g for 30 min and dialysed against 50 mM sodium acetate buffer (pH 5.5). With the AKTA Purifier Plus 10 system (GE, America), Sephacryl S-200HR was used as the medium for size-based column chromatography to isolate the chitinase and the pro-hevein (ProH) protein in B-serum and the hevein protein in the precipitate of 85–95% (NH_4_)_2_SO_4_ saturation. The size-based column was eluted with 50 mM sodium acetate buffer containing 25 mM NaCl (pH 5.5) at a flow rate of 0.2 mL/min, and the sequential fractions of 3 mL were collected. CM Sepharose Fast Flow was used as the medium for cation exchange column chromatography to isolate β-1,3-glucanase in the precipitate of 0–65% (NH_4_)_2_SO_4_ saturation [50]. The column was washed with 50 mM sodium acetate (pH 5.5) and eluted using a linear increasing gradient of NaCl concentration from 0 to 0.5 M at a flow rate of 0.2 mL/min and sequential fractions of 2 mL were collected. The protein concentration of each fraction was measured using the bicinchoninic acid (BCA) assay, and the protein purity was checked by Tricine-SDS-PAGE.

### Protein interaction analysis by gel-filtration column chromatography

The protein interactions were analysed using size-based (gel-filtration) column chromatography [51]. Sephacryl S-200HR medium filled a column that was 70 cm in length and 2.6 cm in diameter (70*2.6 cm) and that was equilibrated with 3–4 bed volumes by neutral washing buffer (50 mM Tris-HCl + 25 mM NaCl, pH 7.2). Clarified B-serum, 2 mL, was loaded on the equilibrated gel-filtration column and eluted with the same buffer at a flow rate of 0.2 mL/min and sequential fractions of 3 mL were collected. The elution peaks were detected by Tricine-SDS-PAGE and western blotting. As a control, clarified B-serum sample was eluted with acidic washing buffer (50 mM sodium acetate+ 25 mM NaCl, pH 5.5).

### Prokaryotic expression, purification, and antibody preparation of actin protein

Total RNA was isolated from latex samples collected from the trunk bark of regularly tapped rubber trees as described by Tang et al. [52]. cDNA was generated using a Reverse Transcriptase kit (TaKaRa, Tokyo, Japan) and used as a template for reverse transcription-PCR (RT-PCR) amplification of the ACTIN gene (Genbank accession No. JF775488) with a pair of specific primers (forward: 5′-cttgCCATGGCCGATGCTGAGGATAT-3′; reverse: 5′-CCGCTCGAGGAAGCACTTCCTGTGAAC-3′) under the following conditions: 40 s denaturation at 98 °C, followed by 30 cycles of amplification (98 °C for 10 s, 60 °C for 30 s, 72 °C for 1 min), and then a final extension for 7 min at 72 °C. The amplified products were added “A” and linked with pMD18-T vectors. The recombinant protein was expressed in *E. coli* BL21 (DE3) after being induced by 0.1 mM isopropyl-β-d-thiogalactoside (IPTG) for 2 h at 37 °C. The recombinant protein was purified by Ni-NTA affinity chromatography (Sangon Biotech Co., Ltd., Shanghai, China) and identified by western blotting. The polyclonal antiserum was produced by subcutaneously injecting SDS-PAGE purified His-tagged actin protein into two New Zealand rabbits (Beijing CoWin Biotech. Co., Ltd., China), and the working dilution ratio was 1: 80,000, as suggested.

### Protein interaction analysis by pull-down

Protein interaction assays were conducted essentially as described using a Pierce™ Pull-Down PolyHis Protein: Protein Interaction Kit (Pierce, America). 150 μg of the purified His-tagged actin (0.26 μg/μL) was diluted to form 600 μL of bait solution by binding buffer and then added to the sterile Pierce Spin Column containing 25 μL of HisPur™ Cobalt Resin (Pierce, America). The mixture of actin bait and the HisPur Cobalt Resin were incubated at 4 °C for at least 30 min, followed by centrifugation at 1250×g for 30–60 s. The bait flow-through solution was collected for subsequent electrophoresis detection. The resin beads were collected and washed to eliminate the nonspecific interactions. The B-serum sample was dialysed and then the clear B-serum dilution solution (0.86 μg/μL) was used as the prey protein sample. 150 μg of the B-serum prey protein was diluted to form 600 μL of prey solution by binding buffer and added into the Pierce Spin Column containing resin beads binding to the polyhistidine-tagged actin bait protein. The mixture was incubated at 4 °C for 60 min with gentle rocking, followed by centrifugation at 1250×g for 30–60 s. The prey flow-through solution was also collected for detection. The pellet of resin beads was washed two times with binding buffer and then incubated for 5 min with 250 μL of elution buffer containing 290 mM imidazole, followed by centrifugation at 1250×g for 30–60 s. The elution solution was collected for Tricine-SDS-PAGE and western blotting.

### Protein interaction analysis by surface plasmon resonance (SPR)

Interactions between β-1,3-glucanase and chitinase, hevein and ProH were analysed by SPR using a Biacore™ 3000 instrument (GE Healthcare, America) at 25 °C. The purified β-1,3-glucanase (1832 μg/mL) was separately diluted to 10–100 μg/mL with 10 mM sodium acetate buffer at pH 5.5, 5.0, 4.5 and 4.0, followed by running the ligand pre-concentration procedure at a flow rate of 10 μL/min to find the superior ligand concentration and a suitable immobilization pH. A CM5 sensor chip (GE Healthcare, America) was activated using a 1:1 ratio of 0.4 M 1-ethyl-3-(3-dimethylamino-propyl)- carbodiimide hydrochloride (EDC) and N-hydroxysuccinimide (NHS) at a flow rate of 5 μL/min, and then β-1,3-glucanase was immobilized on the activated sensor chip by amine coupling. The surface performance test and the regeneration scouting for different analytes were performed separately. Then, individual analytes chitinase, hevein and ProH were flowed over the immobilized β-1,3-glucanase surface at varying concentrations at a flow rate of 30 μL/min with HBS-EP (10 mM HEPES, 150 mM NaCl, 3 mM EDTA, and 0.005% [*v*/v] surfactant P20, pH 7.4) as the running buffer. The contact time was 180 s, and the dissociation time was 300 s. The interactions between the various components were recorded and the curve fitting was analysed with BIA evaluation software (GE Healthcare, America). Rate parameters (ka, kd) and corresponding dissociation constant (KD = kd/ka) were determined by globally fitting all the experimental data to a simple 1:1 L binding model.

### Protein identification analysis

The proteins were identified by matrix-assisted laser desorption/ionization time of flight mass spectrometry (MALDI TOF/TOF MS) according to a reported method [53]. In brief, the collected peptides from trypsin-digested proteins were vacumm-dried, and mass spectra were obtained on an Autoflex MALDI TOF/TOF MS instrument (Bruker Daltonics, Billerica, MA). The spectra were analysed with FlexAnalysis software (Version 3.2, Bruker Daltonics).

## Results

### Identification of the primary components of protein-network at the end of the severed laticifers

**Fig. 1:**
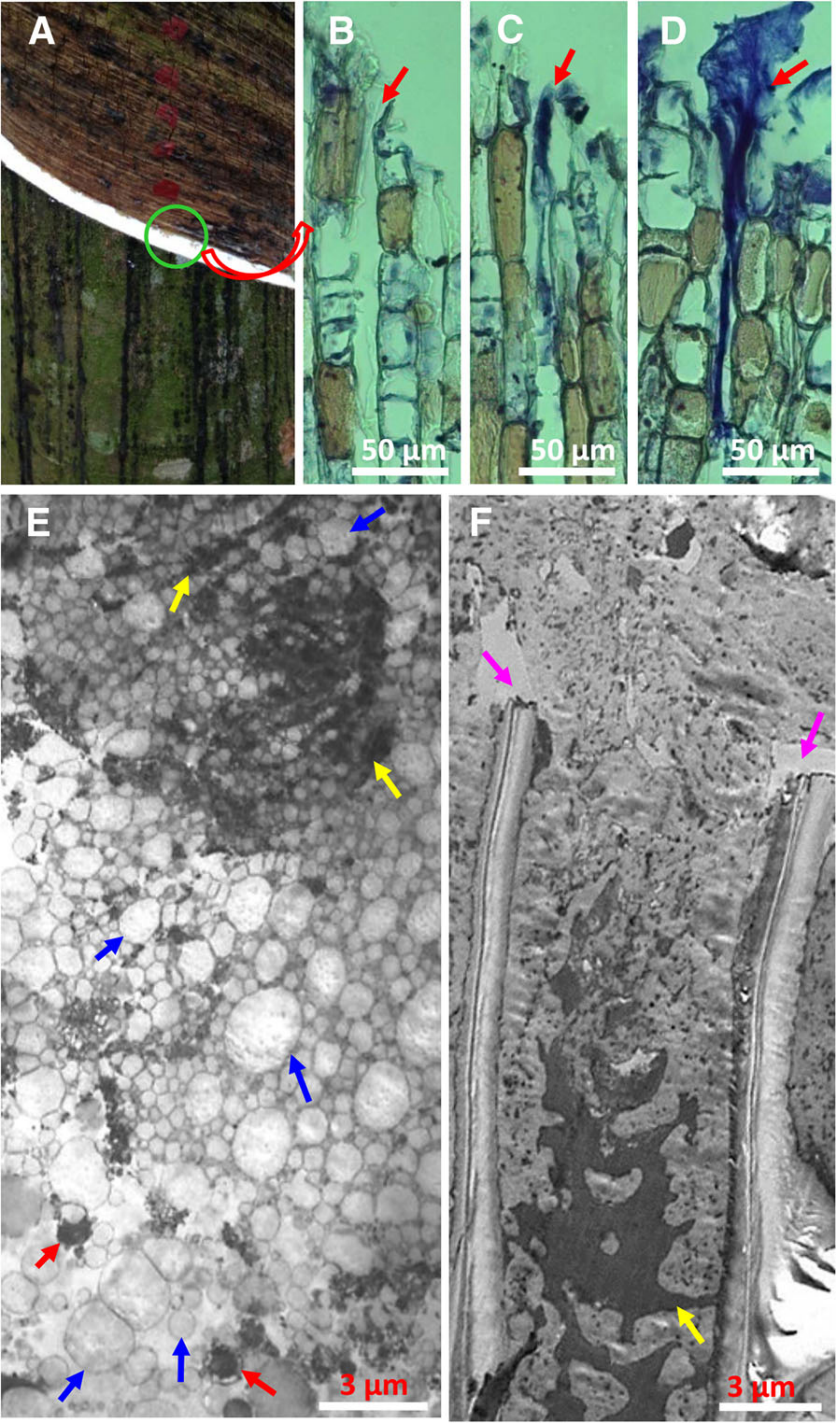
Cytological changes at the end of the severed laticifers after tapping under a light and an electron microscope. **a** The trunk of regularly tapped tree, showing the tapping panel. Green circle shows the site of bark sample collection. **b-d** Radial sections of bark at tapping panel showing the formation of protein-network within and over the laticifer wounds at 0 min (**b**) and 5 min (**c**) after tapping and as soon as the flow of latex stopped (**d**). The red arrow shows the end of the severed laticifers. **e-f** Longitudinal section of the end of the laticifer vessels under an electron microscope, showing the lutoids that changed from an intact vesicle (red arrows) with surrounding membrane to an electron-dense network (yellow arrows), the rubber particles that remained intact (blue arrows) immediately at the cessation of latex flow (**e**), and the coagulated rubber particles without membrane structure and the network of electron-dense materials (yellow arrows) 24 h after latex flow stopped (**f**). The purple arrow shows the cuts of cell wall

Milky latex flowed from the wounded laticifers in bark of rubber tree immediately after tapping (mechanical wounding) (Fig. 1a). No proteinaceous materials were found at the end of the severed laticifers immediately after tapping (Fig. 1b). Five minutes after tapping, sparse proteinaceous materials were observed within the ends of the severed laticifers (Fig. 1c). The proteinaceous materials accumulated gradually as latex flow continued. When the latex flow stopped, the proteinaceous materials were abundant as a network with innumerable fibrils interwoven with one another and arranged roughly paralleling the longitudinal axis of the laticifers (Fig. 1d). The protein-network extended inwards to the severed laticifers approximately 0.7–1.0 mm and outwards to the surface of the tapping panel (Fig. 1d). Under an electron microscope, many electron-dense materials and intact rubber particles were near the end of the severed laticifers soon after the latex flow stopped (Fig. 1e). The electron-dense network-like materials corresponded to the protein-network under a light microscope. Rubber particles with intact membranes aggregated in the spaces among the network materials, whereas intact lutoids were barely detected. The structure of lutoids was intact, with lutoids appearing in a global form with electron-dense inclusions at the site not far from the tapping cut (Fig. 1e). The electron-dense inclusions in the lutoids were likely the source of the electron-dense netlike materials at the end of the severed laticifers, after the burst of lutoids (Fig. 1e). Twenty-four hours after latex flow stopped, rubber coagulum and the network of electron-dense materials were observed at the end of the severed laticifers and extended outside (Fig. 1f).

**Fig. 2:**
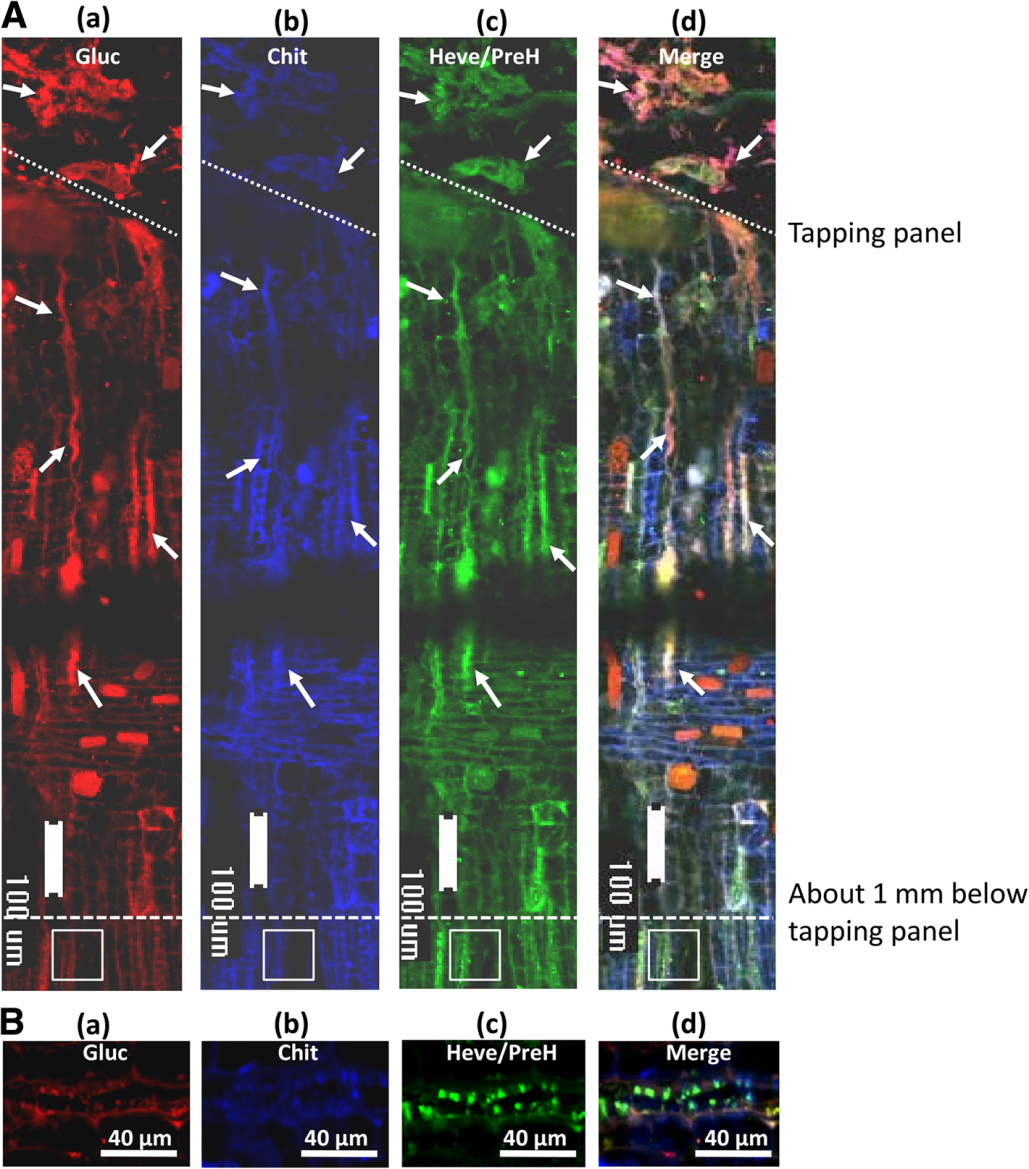
Immuno-histochemical localization of β-1,3-glucanase, chitinase and hevein in the bark at the tapping cuts soon after latex flow stopped. (**A**) The sections were simultaneously treated with polyclonal antibodies raised against β-1,3-glucanase, chitinase and hevein and triply labelled by the secondary RRX-conjugated goat anti-mouse (**a**), AMCA-conjugated goat anti-chicken (**b**), and FITC-conjugated goat anti-rabbit (**c**). (**d**) The merged image of (**a**), (**b**) and (**c**). (**B**) Magnification of the square areas in A. White arrows, protein-network; Gluc, β-1,3-glucanase; Chit, chitinase; Heve, hevein; PreH, the precursor of hevein, including pro-hevein and pre-prohevein

To identify the primary components of the protein-network, immunofluorescent histochemical localization with the polyclonal antibodies of β-1,3-glucanase, chitinase and hevein was performed. In the longitudinal sections of bark at the tapping cut immediately after latex flow stopped, the labelled proteins largely coexisted at the end of the severed laticifers, extending outwards to the tapping panel (Fig. 2A, a-d). The protein-network extended inwards approximately 1 mm where spherical particles with green fluorescence appeared (Fig. 2A, d). The spherical particles were generally less than 8 μm in diameter (Fig. 2B, a-d) and were likely intact lutoids, although red, blue and green fluorescence co-localized in the spherical particles (Fig. 2B, a-c), the spherical particles exhibited green fluorescence in the merged image (Fig. 2B, d), suggesting hevein and its precursor proteins (PreH) were more abundant than chitinase and β-1,3-glucanase.

### Interaction of the primary proteins from the lutoid inclusions

**Fig. 3:**
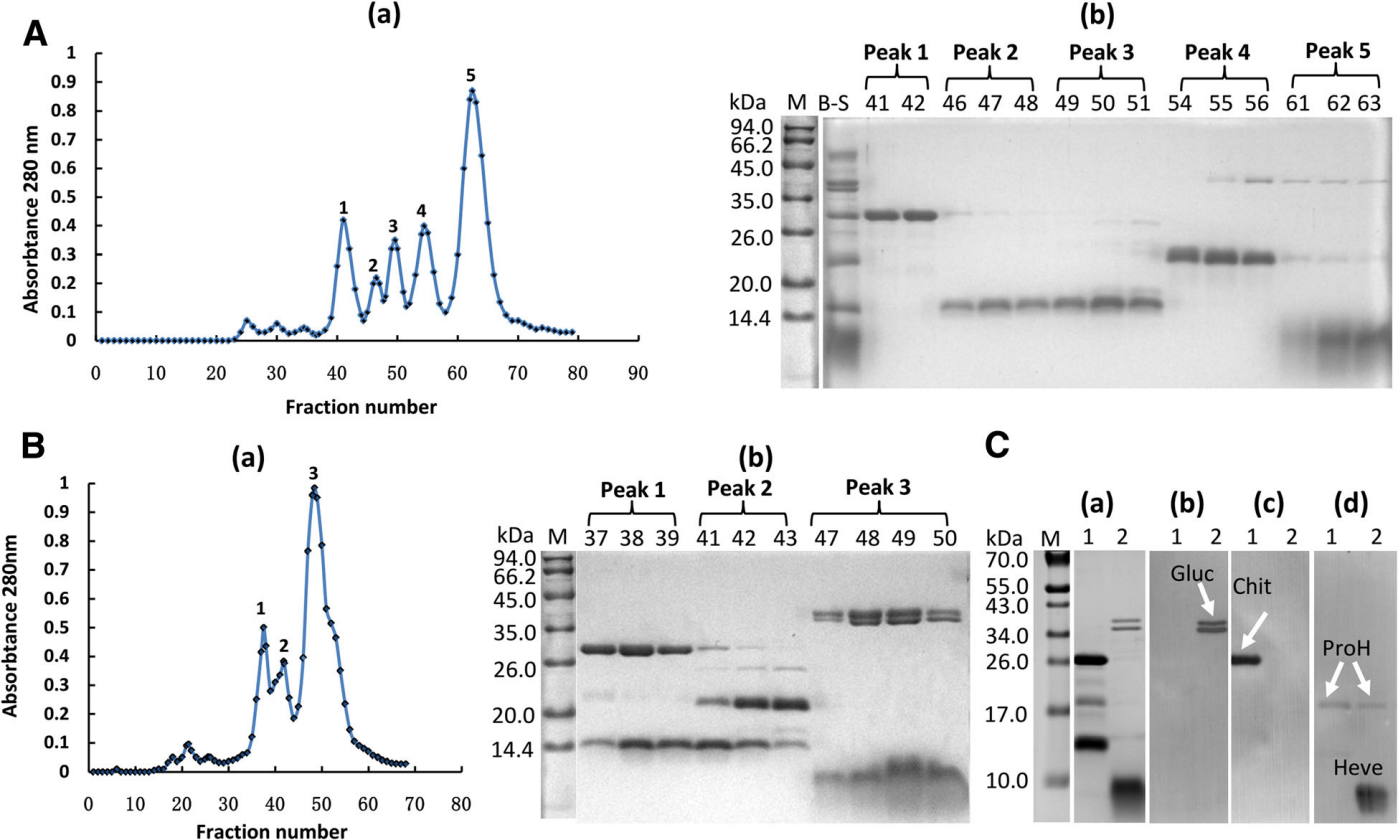
Effect of pH value on the interactions of the primary proteins from B-serum. (**A**) Size-based chromatography profiles of B-serum eluted by acidic washing buffer (50 mM sodium acetate+ 25 mM NaCl, pH 5.5) (**a**), and the protein profiles of Tricine-SDS-PAGE (**b**). (**B**) Size-based chromatography profiles of B-serum eluted by neutral washing buffer (50 mM Tris-HCl + 25 mM NaCl, pH 7.2) (**a**), and the protein profiles of Tricine-SDS-PAGE (**b**). B-S, B-serum. 10 μg of protein was loaded for the B-serum lane. The number above the lane is the fraction number. 10 μL of collection was loaded for each lane. M, protein marker. (**C**) The protein profiles of Tricine-SDS-PAGE (**a**) for the condensed fractions of peak 1 (1) and peak 3 (2) that were obtained by elution with the neutral buffer and western blotting (**b-d**) with the polyclonal antibodies raised against β-1,3-glucanase (**b**), chitinase (**c**) and hevein (**d**). For lanes 1 and 2, 4 and 3 μg of protein was loaded, respectively. M, protein marker; Gluc, β-1,3-glucanase; Chit, chitinase; Heve, hevein; ProH, pro-hevein

A large difference in pH value was observed between the inclusions (i.e., B-serum) (pH 5.5) of lutoids and the lutoid suspended cytosol (pH 6.9) of laticifer cells [27]. Using gel-filtration column chromatography, five primary eluting peaks appeared when the B-serum loaded column was eluted with an acidic washing buffer (50 mM sodium acetate+ 25 mM NaCl, pH 5.5) (Fig. 3A, a). Tricine-SDS-PAGE showed that peak 1 (38–44 fractions) was rich in a 26 kDa protein, peaks 2 and 3 (45–51 fractions) were rich in a 14 kDa protein, peak 4 (52–58 fractions) was rich in a 20 kDa protein and peak 5 (59–68 fractions) was rich in a 10 kDa protein (Fig. 3A, b). A protein with molecular mass of approximately 41 kDa existed simultaneously in peak 4 and peak 5 (Fig. 3A, b). When the B-serum loaded on column was eluted with a neutral washing buffer (50 mM Tris-HCl + 25 mM NaCl, pH 7.2), only three primary eluting peaks were detected (Fig. 3B, a). By Tricine-SDS-PAGE monitoring (Fig. 3B, b), a 26 kDa protein and a 14 kDa protein co-existed in peak 1 (35–39 fractions), a 20 kDa protein and a 14 kDa protein occurred simultaneously in peak 2 (40–44 fractions), and a 35.5 kDa protein, 41 kDa protein and 10 kDa protein were simultaneously eluted in peak 3 (45–56 fractions). The concurrence of proteins with obvious difference in molecular mass undergoing molecular sieve separation suggested interaction among the proteins or existence of multimer. To identify the proteins in peak 1 and peak 3 that were eluted by neutral elution, the proteins in each peak were concentrated for detection by western blotting. The condensed sample from peak 1 contained three primary bands with molecular mass of 26, 20 and 14 kDa in the protein profile of Tricine-SDS-PAGE (Fig. 3C, a1). Three primary protein bands were in peak 3 (Fig. 3C, a2). Based on western blotting, the 35.5 and 41 kDa proteins in peak 3 were β-1,3-glucanase (Fig. 3C, b2), the 26 kDa protein in peak 1 was chitinase (Fig. 3C, c1), the 20 kDa protein in peak 1 and peak 3 was ProH (Fig. 3C, d1–2), and the 10 kDa protein in peak 3 was hevein (Fig. 3C, d2). The 14 kDa protein in peak 1 (Fig. 3C, a1) was not recognized by any of the three types of polyclonal antibodies that were raised against β-1,3-glucanase (Fig. 3C, b1), chitinase (Fig. 3C, c1) and hevein (Fig. 3C, d1). This protein might be the C-terminal of the ProH because available data show that the ProH can be split into C-terminal and N-terminal parts (i.e., hevein) [54]. Hence, interactions among chitinase, ProH and the C-terminal of ProH and those among β-1,3-glucanase, hevein and ProH could occur under the condition of neutral pH.

**Fig. 4:**
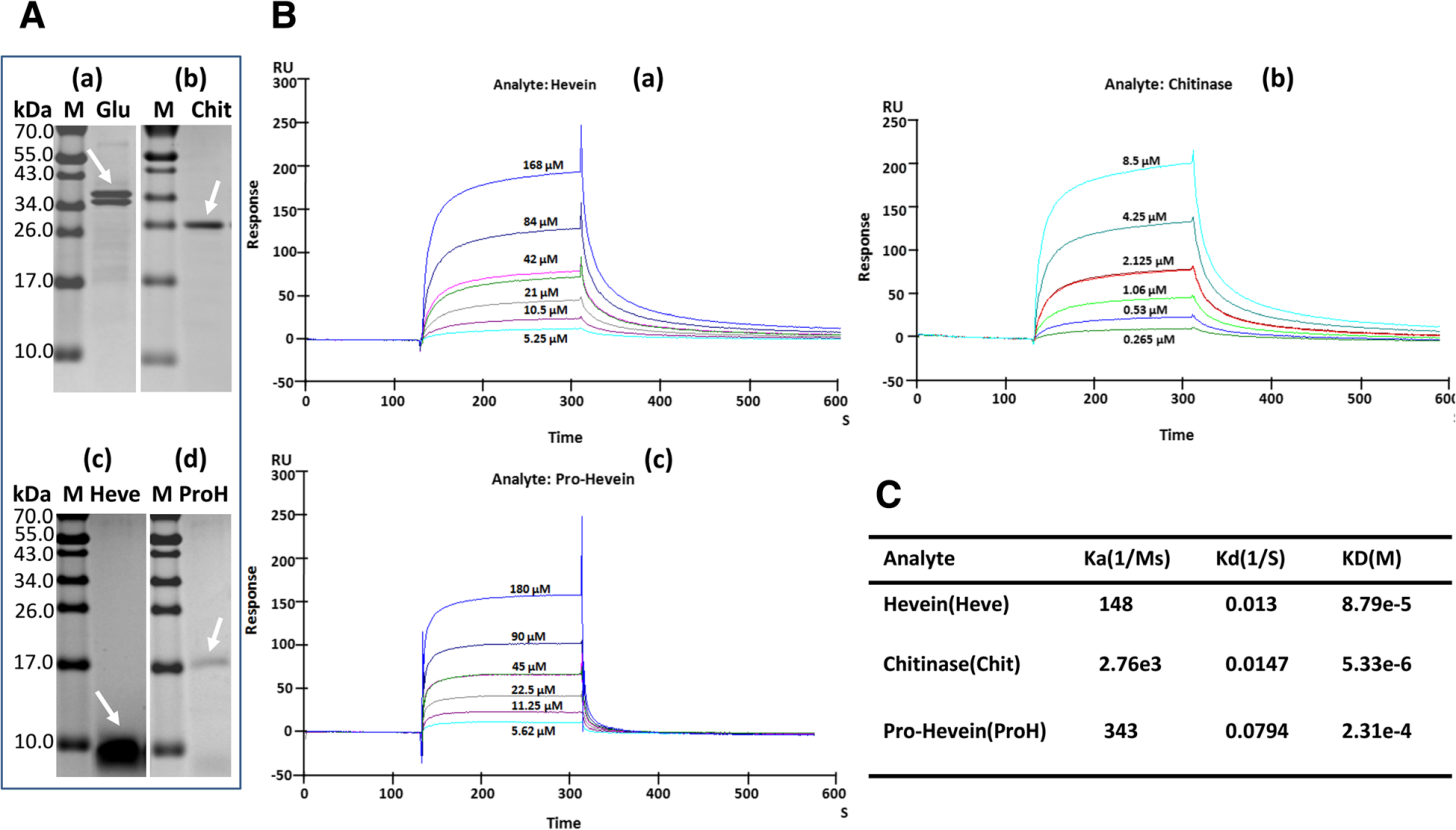
Identification of the interaction of β-1,3-glucanase with chitinase, hevein and pro-hevein by SPR binding response. (**A**) Purity identification of the β-1,3-glucanase (Gluc), chitinase (Chit), hevein (Heve) and pro-hevein (ProH) by Tricine-SDS-PAGE. M, protein marker. Loaded were 1.8 μg of Gluc, 0.8 μg of Chit, 3.2 μg of Heve and 0.37 μg of ProH. (**B**) SPR-based detection of protein binding activity. The binding kinetics of β-1,3-glucanase to hevein (**a**), chitinase (**b**) and pro-hevein (**c**) were obtained by respectively injecting concentrations of hevein ranging from 5.25 to 168 μM, chitinase from 0.265 to 8.5 μM and pro-hevein from 5.62 to180 μM over the β-1,3-glucanase immobilized channel and reference channel at 30 μL/min for 3 min simultaneously. (**C**) The apparent constants Ka (association rate constant), Kd and KD calculated according to the Langmuir binding fitting model

To verify the interactions of the primary proteins from lutoids by the SPR technique, natural β-1,3-glucanase, chitinase, hevein and ProH were purified from B-serum (Fig. 4A). The β-1,3-glucanase was used as ligand to couple with the CM5 chip because of the higher molecular weight and isoelectric point (pI) than those of the other three proteins. Dilution of β-1,3-glucanase with 10 mM sodium acetate buffer (pH 5.0) to 20 μg/mL for a pre-concentration acquired good electrostatic adherence. Amine coupling was used to immobilize the β-1,3-glucanase on the sampling channel. The captured level was 5000 RU. During the flow of individual analytes over the surface of the immobilized β-1,3-glucanase at varying concentrations, the binding cycles were recorded. The regeneration solution was 10 mM glycine at pH 2.5. Hevein was diluted with HBS-EP buffer (10 mM HEPES, 150 mM NaCl, 3 mM EDTA, and 0.005% [*v*/v] surfactant P20, pH 7.4) to a concentration series of 168, 84, 42 (duplicate sample), 21, 10.5 and 5.25 μM for multi-cycle kinetic analysis (Fig. 4B, a). The affinity interaction was medium between β-1,3-glucanase and hevein, considering the dissociation equilibrium constant (*K*_*D*_) was 8.79 e-5 M (Fig. 4C). The chitinase was diluted to an 8.5, 4.25, 2.125 (duplicate sample), 1.06, 0.53 and 0.265 μM series for multi-cycle kinetic analysis (Fig. 4B, b). The *K*_*D*_ was 5.33 e-6 M (Fig. 4C). The pro-hevein also was studied with a 180, 90, 45 (duplicate sample), 22.5, 11.25 and 5.62 μM series for multi-cycle kinetic analysis (Fig. 4B, c). Low-affinity was detected with a *K*_*D*_ of 2.31 e-4 M (Fig. 4C). These results indicated that β-1,3-glucanase interacted with chitinase, hevein and ProH under the condition of HBS-EP buffer at pH 7.4. The highest affinity interaction was for β-1,3-glucanase with chitinase while they were separated by size-based chromatography, which should be attributed to the high sensitivity and the real-time tracking specialty of SPR technology.

### Identification of the interaction of actin with the proteins in B-serum by pull-down

**Fig. 5:**
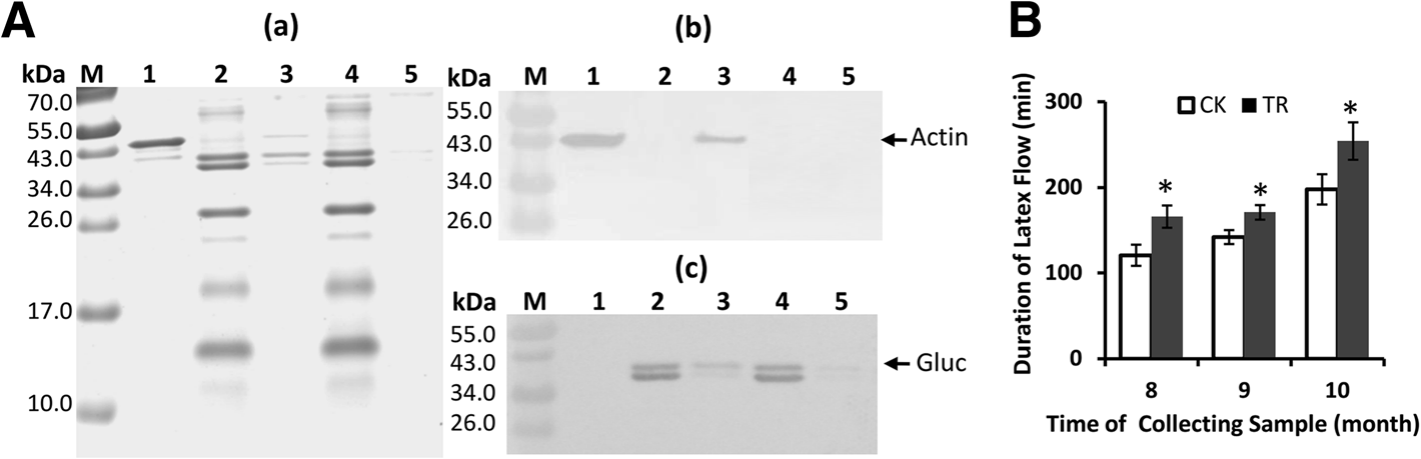
The interaction of actin with β-1,3-glucanase associated with the accumulation of a protein-network. (**A**) The interaction of actin with β-1,3-glucanase was verified by pull-down. (**a**) The Tricine-SDS-PAGE profiles of His-tagged actin (1), the clarified supernatant of B-serum dialysed against TBS buffer (25 mM Tris-HCl + 150 mM NaCl, pH 7.2) (2), the elution solution of the His-tagged actin-binding resin (3), the prey flow-through solution of the His-tagged actin-binding resin (4) and the elution solution of the control (5). Protein was loaded at 1.3, 4.3 and 4.3 μg for lanes 1, 2 and 4, respectively;10 μL of elution solution was loaded for lanes 3 and 5. M, protein marker. (**b-c**) Western blotting analysis of their duplications with polyclonal antibodies raised against His-tagged actin (**b**) and β-1,3- glucanase (**c**). (**B**) The effect of cytochalasin B on the duration of latex flow in the field. TR, treated with dimethyl sulfoxide (DMSO) containing 0.1% cytochalasin B; CK, treated with dimethyl sulfoxide (DMSO) only as the control

The purified His-tagged actin as bait was used to capture the prey proteins in B-serum using pull-down. The purity of His-tagged actin (Fig. 5A, a1) and the primary proteins in B-serum (Fig. 5A, a2) were checked by Tricine-SDS-PAGE. After the binding of the purified His-tagged actin to Cobalt Resin, proteins in the B-serum were incubated with the resin. Then, the resin was washed with binding buffer and eluted with imidazole-containing buffer. Three protein bands were in the Tricine-SDS-PAGE profiles of the elution solution (Fig. 5A, a3), whereas all the primary proteins were in the prey flow-through solution (Fig. 5A, a4). Of note, nearly all the primary proteins in B-serum disappeared in the elution solution when the resin was without binding His-tagged actin (Fig. 5A, a5), suggesting that no specific binding of proteins to the resin occurred. The protein bands in Tricine-SDS-PAGE were identified using antibodies raised against His-tagged actin (Fig. 5A, b) and β-1,3-glucanase (Fig. 5A, c). The polyclonal antibodies that were raised against the His-tagged actin specifically recognized the His-tagged actin (Fig. 5A, b1) and the 40 kDa protein that was eluted from the His-tagged actin-binding resin (Fig. 5A, b3). The polyclonal antibodies that were raised against β-1,3-glucanase specifically recognized two protein bands with molecular mass of approximately 35.5 and 41 kDa in B-serum (Fig. 5A, c2), the elution solution (Fig. 5A, c3) and the prey flow-through solution (Fig. 5A, c4) of His-tagged actin-binding resin. The two bands were weakly detected in the elution solution of the resin without binding of His-tagged actin-binding resin (Fig. 5A, c5), suggesting that a very faint non-specific adherence of β-1,3-glucanase occurred with the resin of control. The protein bands in Tricine-SDS-PAGE also were identified using antibodies raised against hevein and chitinase, but not any bands were detected in the elution solution. The pull-down results indicated the interaction of β-1,3-glucanase with actin, and this interaction might be essential for anchoring the protein-network to actin, contributing to the accumulation of protein-network and formation of the plugs at the end of the severed laticifers during latex flow. The effect of actin on binding the protein-network was further pharmacologically verified in the field. The tapping panel of the regularly tapped rubber trees was treated with 0.1% cytochalasin B (*w*/*v*), a specific reagent for depolymerizing actin [55]. Although the tendency was for the duration of latex flow to increase from August to October under natural conditions, the cytochalasin B treatment resulted in the significant prolongation of the duration of latex flow in each month in comparison with the control (*p* < 0.05) (Fig. 5B). The effect of cytochalasin B on prolonging the duration of latex flow was attributed to hindering the accumulation of protein-network by depolymerizing actin.

### Identification of the proteins that bound to lutoid membrane debris in a pH-dependent manner

**Fig. 6:**
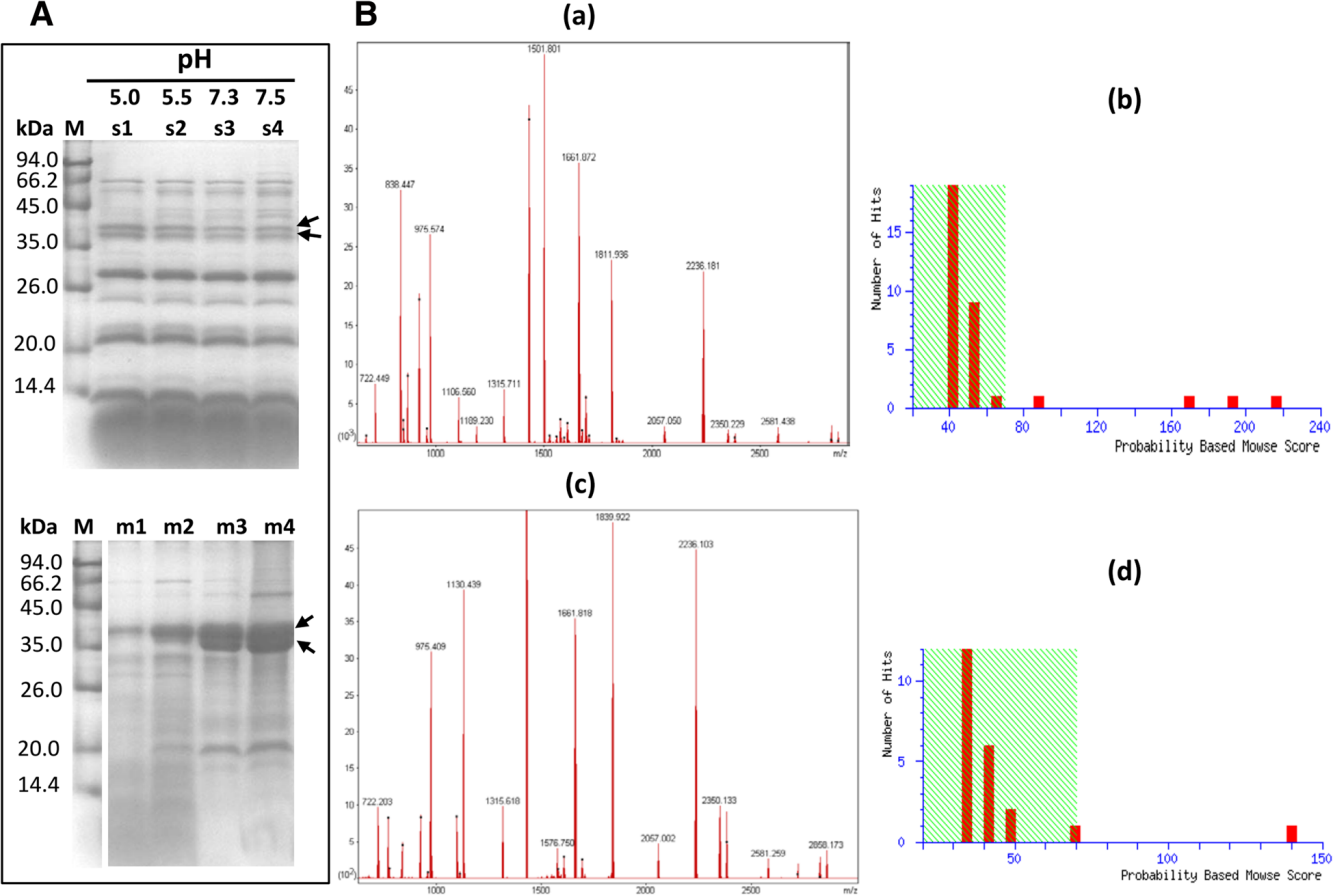
MALDI TOF MS identification of two protein bands with increased level in lutoid membrane debris as pH increased. (**A**) Tricine-SDS-PAGE profiles of soluble protein inclusions (s1-s4) and lutoid membrane debris (m1-m4) when the lutoids were fractured in 50 mM sodium acetate buffer with pH of 5.0 (s1, m1) and 5.5 (s2, m2) and 50 mM Tris-HCl buffer with pH of 7.3 (s3, m3) and 7.5 (s4, m4). (**B**) MALDI-TOF-MS identification, showing the PMF file (**a**) and Mowse score (**b**) of the larger band (arrow) and the PMF file (**c)** and Mowse score (**d**) of the smaller band (arrow)

The clear bottom fraction (lutoids) was respectively re-suspended into four types of buffer: 50 mM sodium acetate buffer with pH of 5.0 and 5.5, and 50 mM Tris-HCl buffer with pH of 7.3 and 7.5. The re-suspensions were subjected to freeze-thaw cycles (− 20 and 37 °C) to release the protein inclusions of lutoids. The supernatants and deposits were collected after centrifugation. In the Tricine-SDS-PAGE profiles, the supernatants and deposits were obviously different (Fig. 6A), and the changes in the level of the two protein bands with a molecular mass between 35 and 45 kDa exhibited a reverse tendency as pH value increased (Fig. 6A). This result indicated that the two proteins in the soluble inclusions of lutoids were largely bound to the lutoid membrane debris when lutoids were fractured in buffers with a neutral pH value. The two proteins were identified as β-1,3-glucanase by MALDI TOF MS (Fig. 6B). The molecular mass of the larger band was 41.543 kDa with a pI of 9.40; whereas the molecular mass of the smaller band was 35.295 kDa with a pI of 9.46.

## Discussion

Laticifers are specialized cells that synthesize and accumulate latex. In the plant kingdom, there are beyond 12,000 species belonging to different families to produce latex. The chemical composition of latex is complex and highly variable in different plant species [29]. The natural rubber in latex as a chemical macromolecule has high economic value and is used widely in various fields. So far, rubber tree is still the most important cultivated plant to provide the commercial natural rubber in the world [27]. In order to harvest the latex of rubber tree, laticifers in the trunk bark must be severed by tapping. The latex flows out from the wound site until the severed laticifers are plugged. The cessation of latex flow is primarily ascribed to the plugs that are formed at the end of the severed laticifers because the latex flow resumes after removing the plugs by tapping [31]. The presence of rubber coagula at the end of the severed laticifers based on electron microscopic observations of Southorn [34] leads to the ordinary viewpoint that the rubber coagula were the plugs. Thereafter, how the rubber particles aggregate to form rubber coagula is investigated and lutoids are generally regarded as the trigger that causes rubber particle aggregation. According to Gidrol et al. [38], hevein, a lectin that is released from the fractured lutoids, is effective in triggering the rubber particle aggregation; whereas chitinase, also released from the fractured lutoids, has an antagonistic effect. Although the chitinase alone inhibits rubber particle aggregation, the combination of chitinase and β-1,3-glucanase enhances the effect of β-1,3-glucanase on rubber particle aggregation [44]. It is difficult to image the chitinase released from lutoid together with other proteins could be isolated spatially and carry out function solely. Factors from the fractured lutoids should overally trigger the rubber particle aggregation in situ. And moreover, the role of lutoids might not only as a trigger because of their 10–20% volume in the latex.

Instead of rubber coagula, it is the protein-network and aggregates of intact rubber particles that are present at the end of the severed laticifers soon after the latex flow stopped (Fig. 1, e), which is consistent with our previous observation [42]. The rubber coagula appeared much later than the appearance of protein-networks and rubber particle aggregates (Fig. 1, f), suggesting that the formation and accumulation of protein-networks and rubber particle aggregates other than rubber coagula are associated with the initial cessation of latex flow. The primary components of the protein-networks are the major protein inclusions that were released from the fractured lutoids as revealed by triple immuno-fluorenscence localization. It is noted that the lutoids are defined as polydispersed lysosomal vacuoles with a pH of 5.5–6.0 [35, 56] and are suspended in the cytosol of laticifer cells where the pH is 6.5–7.3 [57]. The difference in the pH value between the inclusion of lutoids and the cytosol of laticifer cells seems crucial for the formation of protein-networks when the proteins are released from the fractured lutoids into the cytosol of the laticifer cells. Under a neutral pH condition like the case of the cytosol of laticifer cells, the interactions of lutoid-originated proteins occur as revealed by gel-filtration column chromatography and SPR technique (Figs. 3 and 4).

The turgor pressure of laticifers in a rubber tree reaches up to 10–15-fold of atmospheric pressure before tapping [58, 59]. The high pressure is the driving force to push the latex out of the severed laticifers after tapping. It thus should hinder the accumulation of the aggregates of rubber particles caused by either hevein [38] or lutoid membrane debris [37], and PPO [41] and the protein-network [42] at the end of the severed laticifers. However, the protein-network can be accumulated soon after tapping (Fig. 1, c). We previously observed actin accumulates at the end of the severed laticifers during latex flow [60]. It is a reasonable suggestion that the binding of protein-network to actin may be associated with the soon accumulation of protein-network at such site. In the present study, the interaction of the lutoid-originated β-1,3-glucanase with actin was demonstrated by pull-down analysis and pharmacologically verified by cytochalasin B—caused significant prolongation of the duration of latex flow in the field.

The accumulation of protein-network by the interaction of the primary component β-1,3-glucanase with actin is essential for plug formation. For one thing, rubber particles could bind to the protein-network because β-1,3-glucanase, hevein and the combination of β-1,3-glucanase and chitinase is effective in aggregating rubber particles [38, 44]. For the other, the lutoid membrane debris with the aggregates of rubber particles [37, 61] could also adhere to the protein-network by binding of the debris to β-1,3-glucanase (Fig. 6). In this way, the large and tight plugs are formed by binding of the lutoid debris and rubber particles to the protein-network, resulting in the rapid occlusion of the severed laticifers. Furthermore, PPO may also participate in the plugs by binding of rubber particles [40, 41]. The protein-network was also a biochemical barrier to protect the wounded laticifer cells from pathogen invasions given that the primary components of protein-network, β-1,3-glucanase, chitinase and hevein, possess antifungal activity [21, 49, 62, 63], which was consistent with the concept of latex as a plant defense system [64]. The accumulation of antimicrobial proteins and actin at a wound site may be a universal response of wounded cells. An actin reticulum is also discovered throughout the cytoplasm of coenocytic green algae cells shortly after wounding [65]. Available data show that the binding of chitinase and osmotin-like proteins to actin filaments likely participated in potato cell defence against pathogen attack [66] and the actin cytoskeleton is required for plant system immunity [67–69].

In summary, the decrease in the turgor pressure results in lutoid burst at the end of the severed laticifers during latex flow after tapping. The protein inclusions such as β-1,3-glucanase, chitinase and hevein are released from the fractured lutoids into cytosol of laticifer cells. The change in pH value from acetic (5.5–6.0) to neutral (6.5–7.3) causes the physical interaction of these lutoid-originated proteins to form a protein-network. The protein-network accumulates by binding of the primary component, β-1,3-glucanase, to actin filaments. The binding of lutoid membrane debris and rubber particle aggregates to the accumulated protein-network forms a large and tight plug and results in the rapid occlusion of the severed laticifer. The protein-network in combination with the actin filaments at the wounded site of laticifer cells provides not only a physical barrier to stop the loss of latex, the cytoplasm of laticifer cells, but also a biochemical barrier to protect the wounded laticifer cells from pathogen invasion.

## Conclusions

The results suggest that the formation of protein-network by interactions of the proteins with anti-pathogen activity released from lutoids and accumulation of protein-network by binding to the cytoskeleton are crucial for the rapid occlusion of laticifer cells in rubber tree. The protein-network at the wounded site of laticifer cells provides not only a physical barrier but also a biochemical barrier to protect the wounded laticifer cells from pathogen invasion.

## Abbreviations

AMCA: Aminomethylcoumarin acetate
AP: Alkaline phosphatase
BCA: Bicinchoninic acid
BSA: Bovine serum albumin
Chit: Chitinase
DMSO: Dimethyl sulfoxide
EDC: 1-ethyl-3-(3-dimethylamino-propyl) -carbodiimide hydrochloride
FITC: Fluorescein isothiocyanate
Gluc: β-1,3-glucanase
Heve: Hevein
KLH: Keyhole limpet hemocyanin
MALDI TOF/TOF MS: Matrix-assisted laser desorption/ionization time of flight mass spectrometry
NHS: N-hydroxysuccinimide
OGs: Oligogalacturonides
PPO: Polyphenoloxidase
PreH: The precursor of hevein, including pro-hevein and pre-prohevein
ProH: Pro-hevein
PVDF: Polyvinylidene fluoride
RRX: Rhodamine Red-X
RT-PCR: Reverse transcription-PCR
SPR: Surface plasmon resonance
Tricine-SDS-PAGE: Tricine-sodium dodecyl sulphate-polyacrylamide gel electrophoresis
